# An Approach to measuring Integrated Care within a Maternity Care System: Experiences from the Maternity Care Network Study and the Dutch Birth Centre Study

**DOI:** 10.5334/ijic.2522

**Published:** 2017-06-20

**Authors:** Inge C. Boesveld, Pim P. Valentijn, Marit Hitzert, Marieke A.A. Hermus, Arie Franx, Raymond G. de Vries, Therese A. Wiegers, Marc A. Bruijnzeels

**Affiliations:** 1 Jan van Es Institute, Netherlands Expert Centre Integrated Primary Care, Almere, NL; 2 Division Woman and Baby, University Medical Centre Utrecht, Utrecht, NL; 3 Department of Health Services Research, Medicine and Life Sciences, Faculty of Health, Maastricht University, Maastricht, NL; 4 Department of Obstetrics and Gynaecology, Erasmus University Medical Centre, Rotterdam, NL; 5 Department of Child Health, TNO, PO Box 2215, 2301 CE Leiden, NL; 6 Department of Obstetrics, Leiden University Medical Center, PO Box 9600, 2300 RC Leiden, NL; 7 Midwifery Practice Trivia, Werkmansbeemd 2, 4907 EW Oosterhout, NL; 8 Academie Verloskunde Maastricht/Zuyd University, CAPHRI School for Public Health and Primary Care, Maastricht, NL; 9 NIVEL, Netherlands Institute for Health Services Research, Utrecht, NL

**Keywords:** integrated care, questionnaire, psychometric properties, validity, birth care

## Abstract

**Introduction:**

Integrated care is considered to be a means to reduce costs, improve the quality of care and generate better patient outcomes. At present, little is known about integrated care in maternity care systems. We developed questionnaires to examine integrated care in two different settings, using the taxonomy of the Rainbow Model of Integrated Care. The aim of this study was to explore the validity of these questionnaires.

**Methods:**

We used data collected between 2013 and 2015 from two studies: the Maternity Care Network Study (634 respondents) and the Dutch Birth Centre Study (56 respondents). We assessed the feasibility, discriminative validity, and reliability of the questionnaires.

**Results:**

Both questionnaires showed good feasibility (overall missing rate < 20%) and reliability (Cronbach’s Alpha coefficient > 0.70). Between-subgroups post-hoc comparisons showed statistically significant differences on integration profiles between regional networks (on all items, dimensions of integration and total integration score) and birth centres (on 50% of the items and dimensions of integration).

**Discussion:**

Both questionnaires are feasible and can discriminate between sites with different integration profiles in The Netherlands. They offer an opportunity to better understand integrated care as one step in understanding the complexity of the concept.

## Introduction

Integrated care is increasingly promoted for people with complex needs in high-income countries. Their health care systems are facing a variety of inter-related challenges, including: the growing demand for health services; fragmentation of services; changing health needs; and the increasing influence of economic, political, and social factors on health care delivery. Based on evidence, policymakers facing these challenges are turning to “integrated care” as a way to reduce costs, improve the quality of care, and generate better patient outcomes [1,2,3,4]. Integrated care is also increasingly promoted in the Dutch maternity care system [5,6]. A better understanding of integration in perinatal care is, therefore, desirable.

There are many variations in how to organize perinatal care throughout the industrialized world. In the Netherlands, an important feature of the maternity care system is a clear distinction between the first echelon (midwife-led, community based) and second echelon (obstetrician-led, hospital based) [7,8]. The Dutch system is founded on the notion that pregnancy, birth and puerperium are primarily physiological processes. Most pregnant women are considered to be healthy (‘low risk’) and, therefore, receive antenatal care from a community midwife from the beginning of their pregnancy [9]. When complications arise or become threatening, or pharmacological pain relief is requested, referral to secondary or tertiary specialist care (i.e. obstetricians) is necessary [7,8]. Community midwives are independently operating professionals, working in their own midwifery practices in the community. Their position in the health system is comparable to that of general practitioners as gatekeepers to specialist care [10]. Secondary and tertiary obstetricians are mostly organized by partnerships and are working in hospitals. Professionals at all care levels work autonomously and play complementary roles [11].

In recent years, the Dutch maternity care system has come under pressure as a result of the Euro-Peristat studies, which concluded that the perinatal mortality rates in the Netherlands were relatively high as compared to other European countries [5]. In spite of questions about the comparability of the data, concerns about the Dutch maternity care system have been high on the political agenda, with outcomes being linked directly to the organization of this system.

In 2009, a ministerial steering committee installed by the Ministry of Health published a report suggesting improvements in the Dutch maternity care system. Their report stated that the system needs to be ‘effective’, ‘safe’ and ‘patient-centred’ [6]. Based on the assumption that more integrated care could provide higher quality of care, the committee suggested that a possible way to achieve this is by improving collaboration between primary and secondary care through increased integration in both birth centres and existing regional Maternity care Collaboration and Consultation Groups, called Maternity care networks in this paper. Members of these networks include community midwives and obstetricians along with (depending on the regional situation) clinical midwives, paediatricians, managers of maternity care assistance organizations, obstetrics and gynaecology nurse specialists, and general practitioners [12].

Integrated care refers to a co-ordinated and coherent set of services that are planned, managed, and delivered to individual service users across several organizations and co-operating professionals [13,14]. The essence of integrated care is a continuum of care for service users, which crosses the boundaries of public health, primary, secondary, and tertiary care [3,15,16]. At present, little is known about integrated care in the Dutch maternity care system, and no evidence exists supporting the assumption that integrated care improves the quality of birth care. While many evaluations of collaboration in these systems have been conducted (e.g. [17,18,19,20,21,22,23,24,25]), most of these evaluations solely focus on collaboration between professionals and lacks a focus on collaboration between organizations. In 2013, based on the Rainbow Model of Integrated Care [10], an instrument was developed to describe levels of integration in birth care settings, resulting in questionnaires to explore integrated care in birth centres and in Maternity care networks. These questionnaires were used in the Dutch Birth Centre Study [9] and the Maternity care network Study [26]. The aim of the present study is to explore the validity of the Dutch Birth Centre Integration Questionnaire (DBC-IQ) and the Maternity care networks Integration Questionnaire (MCN-IQ), in order to determine whether the questionnaires are useful to measure integration in a maternity care system.

## Theory and methods

### Theoretical background

**Figure 1:**
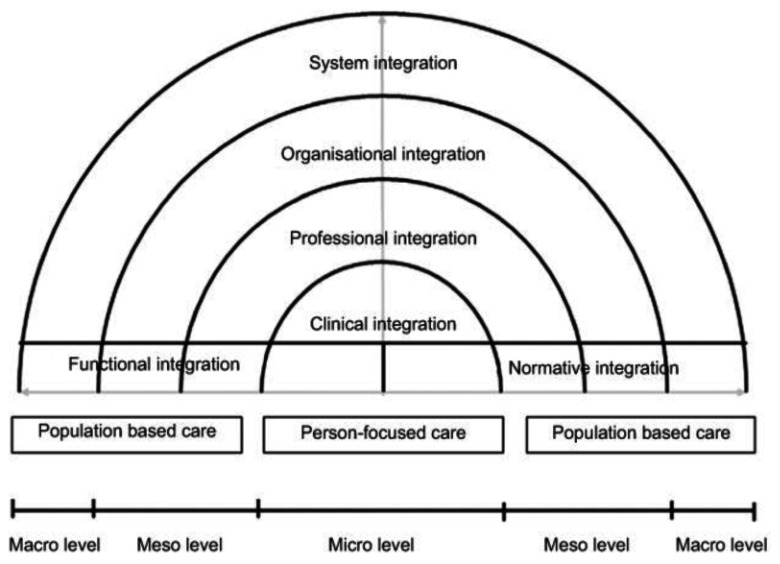
Rainbow Model of Integrated Care. Adopted with permission from: “Understanding integrated care: a comprehensive conceptual framework based on the integrative functions of primary care” (10).

The Rainbow Model of Integrated Care was developed to obtain a better understanding of the concept of integrated care from a primary care perspective [10] (see Figure 1). This conceptual framework combines dimensions of integrated care with the organization and functions of primary care. The model includes multiple dimensions of integration that play complementary roles. It distinguishes four dimensions on the micro, meso and macro levels (clinical, professional, organizational and system integration) to deliver comprehensive services that address the needs of individual people and the population. It also distinguishes two dimensions – functional and normative integration – to ensure connectivity between the levels. The Rainbow Model of Integrated Care is considered useful to understand the complex and multidimensional nature of integrated care [27]. The model is specified in a taxonomy consisting of 59 determinants, based on a literature review and a Delphi study among Dutch experts, validated by expert panels in international conferences held in Singapore and Brussels [16,28]. Due to the characteristics of the Dutch maternity care system, the Rainbow Model of Integrated Care can be used to evaluate birth care in different settings. Therefore, the taxonomy afforded by the model [16] was used to develop two questionnaires to ascertain a better understanding of integrated care birth care settings.

### Methods

#### Development of the questionnaires

The starting point for creating our questionnaires was a survey used to examine integrated care in primary care organizations [29]. For each dimension of integration in the Rainbow Model of Integrated Care, we identified determinants of integration (items). The inclusion procedure for these items was based on the following conditions: highest panel median in a Delphi study [16] and applicability in birth (centre) care. For each item, we formulated answer categories that corresponded with stages of integration: from one (not integrated) to four (fully integrated), forming a nominal scale, with equal weight between the answer categories. The equal weighting was an assumption because we had no way of knowing whether the distances between the answers were regarded as equal by the respondents. Statements corresponding to each stage were derived from the primary care questionnaire and birth centre practice [29]. The questionnaires were tested in a pilot study by three community midwives familiar with birth (centre) care and we adapted some questions/statements based on their comments.

We first constructed the MCN-IQ, which consisted of 20 questions with two to four questions for each dimension. The aim of this questionnaire was to present professionals of Maternity care networks a way to reflect on their level of network integration in order to support their efforts to improve collaboration. Based on our experiences of using this questionnaire, we then constructed the DBC-IQ. The aim of this questionnaire was to classify birth centres in groups with similar integration profiles, as a necessary first sept for outcome evaluation. To create a balanced questionnaire, we formulated the same number of questions for each dimension of integration. Therefore, this questionnaire consisted of 24 questions, and 19 items are the same in both questionnaires. Formulations of the questions were adapted to the settings.

Table 1 reports the items and dimensions of integration used in both questionnaires.

**Table 1:** Integrated care dimensions and used items of the Rainbow Model of Integrated Care in the MCN-IQ and DBC-IQ.*

Level	Dimension	Description dimension	Determinant MCN-IQ	Determinant DBC-IQ	Description determinant

Micro	Clinical integration	The coordination of person-focused care in a single process across time, place and discipline.	Case management	Case management	Coordination of care for clients’ with a high-risk profile (e.g. identifying risks, developing policies and guidance).
			Continuity	Continuity	The organization of care is aimed to provide fluid care delivery for an individual client.
			Individual multidisciplinary care plan	Individual multidisciplinary care plan	Implementation of a multidisciplinary care plan at the individual client level.
			Client participation	Client participation	Clients are (pro) actively involved in the design, organization and provision of care at the operational level.
Meso	Professional integration	Inter-professional partnerships based on shared competences, roles, responsibilities and accountability to deliver a comprehensive continuum of care to a defined population.	Inter-professional education	Inter-professional education	Inter-professional education for professionals focused on interdisciplinary collaboration.
			Shared vision between professionals	Shared vision between professionals	A shared vision between professionals focused on the content of care.
			Multidisciplinary guidelines and protocols	Multidisciplinary guidelines and protocols	Multidisciplinary guidelines and protocols are implemented coherently at the operational level.
			Inter-professional governance	Inter-professional governance	Inter-professional governance focused on openness, integrity and accountability between professionals at the operational level (e.g. joint accountability, appeal on pursued policies and responsibilities).
Meso	Organizational integration	Inter-organizational relationships (e.g. contracting, strategic alliances, knowledge networks, mergers), including common governance mechanisms, to deliver comprehensive services to a defined population.	Performance management	Performance management	Collective elaborated performance management between organizations within the collaboration.
			Learning organisations	Learning organisations	Collective learning power between the organizations within the collaboration (e.g. joint research and development programs).
			Complaints procedure	Complaints procedure	Existing complaints procedure for the partnership.
				Interest management	A climate that attempts to bridge the various interests (e.g. social, organizational and personal interests) at the operational, tactical and strategic level.
Macro	System integration	A horizontal and vertical integrated system, based on a coherent set of (informal and formal) rules and policies between care providers and external stakeholders for the benefit of people and populations.	Stakeholder management	Stakeholder management	Engagement of various stakeholders (e.g. municipality, patient organizations and health insurance company).
			Environmental climate	Environmental climate	Political, economic and social climate in the environment of the collaboration (e.g. market characteristics, regulatory framework, competition).
				Available resources	Available resources in the environment of the collaboration (e.g. usable buildings, (over) capacity, professionals and funding streams).
				Good governance	Creating trust towards external stakeholders (e.g. municipality and health insurance company) due to working method, reputation, management, control and/or supervision.
Micro, meso, macro	Functional integration	Key support functions and activities (i.e. financial, management and information systems) structured around the primary process of service delivery, to coordinate and support accountability and decision making between organizations and professionals to add overall value to the system.	Information management	Information management	Aligned information management systems accessible at operational, tactical and strategic levels (e.g. monitoring and benchmarking systems).
			Service management	Service management	Aligned service management for the client (e.g. collective telephone number, counter assistance and 24-hour access).
			Regular feedback of performance indicators	Regular feedback of performance indicators	Regular feedback of performance indicators for professionals at the operational level to enable them to improve their performance.
				Resource management	Coherent use of resources (e.g. collective real estate and funding).
Micro, meso, macro	Normative integration	The development and maintenance of a common frame of reference (i.e. shared mission, vision, values and culture) between organizations, professional groups and individuals.	Experienced trust		The extent to which those involved in the collaboration at operational, tactical and strategic levels experience trust from their partners.
			Visionary leadership	Visionary leadership	Leadership based on a personal vision that inspires and mobilizes people.
			Quality features of the informal collaboration	Quality features of the informal collaboration	Effectiveness and efficiency of the informal collaboration at the operational, tactical and strategic levels (e.g. group dynamics and attention to the undercurrent).
			Trust	Trust	The extent to which those involved in the collaboration at operational, tactical and strategic levels trust each other.
				Reliable behaviour	The extent to which the agreements and promises within the collaboration are fulfilled at operational, tactical and strategic levels.

Adapted with permission from: “Towards a taxonomy for integrated care; a mixed-methods study” [28]. * Both questionnaires are available in English and Dutch (see attached files). Researchers are allowed to use the questionnaires for research purposes after permission of the author.

#### Study population

##### Maternity Care Network Study: MCN-IQ

In 2013 and 2014, information meetings about models of integrated birth care and related finance were organized for regional Maternity Care Networks in the Netherlands. These meetings aimed to: 1) inform professionals about proposed changes in the organization of birth care by the Dutch government and about implications of these changes for their organizations and 2) allow professionals to reflect upon the level of their network integration, based on the MCN-IQ. The aim of this reflection was to support the networks to improve their collaboration. All over the country, these networks were invited to hold one of these information meetings in their region. Three weeks before the meeting, the MCN-IQ was send by e-mail to the members of the Maternity care networks. The number of questionnaires varied from 20 to 125 per network, depending on the network’s size. Overall 813 participants returned the list (a response rate of 53%). Most of the respondents were community midwives (48%), followed by clinical midwives (14%), obstetricians (12%), managers of maternity care assistance organizations (8%) and paediatricians (5%). 179 respondents (22%) did not complete more than 30% of the items and were excluded, resulting in 634 questionnaires being suitable for analysis.

##### Dutch Birth Centre Study: DBC-IQ

The Dutch Birth Centre Study was designed to present evidence-based recommendations for the organization and functioning of future birth centres in the Netherlands, based on the careful assessment of existing birth centres (9). Based on the definition of birth centres, 23 birth centres were identified at the reference date (September 2013) [Hermus et al., 2016]. These centres included relatively new birth centres and those with a longer history, mono-disciplinary- and multidisciplinary-orientated birth centres, and birth centres with different histories of development. All 23 centres were included in our study and invited to participate, and all the managers gave their permission for their birth centre to participate. Subsequently, we asked managers of birth centres to select two or three care providers from different professions working within or with the birth centre to be interviewed. Depending on the local situation, those invited to be interviewed included: community midwives, maternity care assistants, clinical midwives, obstetric nurse specialists and obstetricians. The researcher (IB) contacted all participants to explain the study. Two weeks before the visits and interviews, the DBC-IQ was sent to the manager and professionals of each birth centre by e-mail. One week later a reminder was sent to any non-responders. Between January 2014 and April 2015, all 23 birth centres participated in this study. These birth centres were located throughout the Netherlands in both urban and rural areas. We sent 73 questionnaires to managers and professionals of birth centres (range 2–5) and 61 of them opened the online questionnaire (a response rate of 84%). Five respondents (8%) failed to complete more than 30% of the items and these responses were excluded, resulting in 56 questionnaires suitable for the analyses.

### Data analysis

We evaluated the following psychometric properties of the MCN-IQ and DBC-IQ: feasibility, discriminatory validity and reliability. To determine the feasibility of both questionnaires, we calculated the missing item rates per dimension of integration and the maximum rate per item. While we found few recommendations in the literature for a cut off point for acceptable response rates for surveys, we determined that missing item rates below 20% were acceptable [30,31]. To assess the discriminative validity of the questionnaires, we took two steps to calculate the integration scores per Maternity Care Network and birth centre. First we calculated the mean scores on the items and per dimension for each respondent (range 1–4). Secondly, we calculated the mean scores of all respondents per item and on the six dimensions of integration for each Maternity Care Network and birth centre (range 1–4). In addition, we computed the total integration score in both settings using the mean score over the six dimensions (range 1–4). To examine the differences between Maternity Care Networks and birth centres on the items and dimensions of integration and the total integration score, we performed a between-subgroup post-hoc test, using a one-way analysis of variance (ANOVA).

To verify in a qualitative way the ability of the MCN-IQ to discriminate between organizations, we asked persons familiar with Maternity Care Networks to nominate the most and least integrated groups for the first eight Maternity Care Networks that participated in the study. We made sure that they did not know the results of the MCN-IQ when ranking the Maternity Care Networks When presenting the results during the information meetings about models of integrated birth care and related finance, we asked the participants of the Maternity Care Networks the extent to which they recognised the results of the assessments.

To assess the reliability of both questionnaires, we calculated the internal consistency by using Cronbach’s alpha coefficient for both the total questionnaires and the six dimensions of integration. Alpha coefficients above 0.70 were considered an adequate indication of internal consistency [30]. To examine the consistency of the answers given by the respondents of each Maternity Care Network, we calculated the range of mean scores on the integration dimensions. We also determined the difference in mean scores of primary care () and secondary care professionals in each Maternity Care Network. To do so, we classified community midwives, general practitioners and (managers of) maternity care assistants as “primary care professionals” and clinical midwives, obstetricians, obstetrics and gynaecology nurse specialists, managers of hospitals as “secondary care professionals”. This consistency analysis was not possible for the DBC-IQ because of the small number of questionnaires. All data analyses were performed using SPSS version 22 (IBM Statistics).

## Results

### Maternity Care Network Study: MCN-IQ

The average item missing rate of the MCN-IQ was 9% (1180 of 12960 items). Maximum item missing rates per dimension ranged from 8.8 to 11.6%. The highest missing rate was on the functional dimension. All missing rates were below the predefined threshold of 20% (see Table 2).

**Table 2:** Missing item values and the maximum percentage missing per item for each dimension of integration for MCN-IQ and DBC-IQ.

Dimension	MCN-IQ (n = 707)				DBC-IQ (n = 58)			

	Total items	Missing items (n)	Missing per domain (%)	Missing per item; maximum (%)	Total items	Missing items (n)	Missing per domain (%)	Missing per item; maximum (%)

**Clinical integration**	2532	296	12	11.1	222	10	5	6.9
**Professional integration**	2659	169	6	8.8	223	9	4	6.9
**Organizational integration**	1943	178	9	10.9	223	9	4	6.9
**Functional integration**	1923	198	10	11.6	220	12	5	13.8
**System integration**	1317	97	7	10.9	214	18	8	12.1
**Normative integration**	2586	242	9	9.3	224	8	4	5.2
**Total**	12960	1180	9	10.4	1326	66	5	8.6

Table 3 presents the mean scores for each Maternity Care Network on the dimensions of integration and the total integration scores.

**Table 3:** Integration characteristics of Maternity Care Networks.

Maternity Care Collaboration and Consultation Groups Study																
			CI		PI		OI		FI		SI		NI		TI	
			
VSV	N	Range	M	SD	M	SD	M	SD	M	SD	M	SD	M	SD	M	SD

1	32	1–4	2.05	0.46	2.35	0.67	2.14	0.55	1.43	0.37	1.75	0.71	2.80	0.51	2.09	0.38
2	30	1–4	1.98	0.53	2.29	0.59	1.97	0.39	1.63	0.52	2.13	0.56	2.79	0.45	2.13	0.33
3	13	1–4	1.73	0.31	2.53	0.60	2.12	0.43	1.21	0.29	1.69	0.60	2.85	0.55	2.02	0.33
4	27	1–4	2.27	0.53	2.37	0.59	1.94	0.37	1.43	0.45	2.33	0.65	2.88	0.48	2.20	0.30
5	23	1–4	1.80	0.31	2.41	0.58	2.02	0.25	1.46	0.44	1.98	0.80	2.55	0.61	2.04	0.36
6	18	1–4	1.95	0.47	2.81	0.59	2.30	0.27	1.78	0.44	2.61	0.37	3.06	0.36	2.42	0.21
7	24	1–4	2.18	0.47	3.02	0.77	2.61	0.46	2.17	0.48	2.56	0.66	3.16	0.56	2.62	0.40
8	13	1–4	1.92	0.48	1.58	0.44	1.67	0.70	1.26	0.31	1.65	0.66	2.47	0.46	1.76	0.33
9	27	1–4	2.18	0.35	2.77	0.49	2.43	0.43	1.74	0.52	2.59	0.64	3.07	0.50	2.46	0.34
10	35	1–4	1.59	0.42	2.50	0.49	2.18	0.37	1.44	0.38	2.40	0.71	2.71	0.53	2.13	0.26
11	35	1–4	1.69	0.38	1.92	0.51	1.92	0.40	1.21	0.28	1.36	0.51	2.60	0.59	1.78	0.29
12	28	1–4	2.46	0.57	2.73	0.63	2.32	0.51	1.76	0.57	2.07	0.73	3.17	0.51	2.42	0.42
13	50	1–4	1.74	0.33	2.17	0.57	2.00	0.39	1.46	0.42	1.45	0.52	2.52	0.47	1.89	0.29
14	32	1–4	1.67	0.36	1.82	0.47	1.83	0.50	1.19	0.26	1.91	0.50	2.41	0.52	1.81	0.27
15	15	1–4	1.79	0.43	2.02	0.71	1.97	0.56	1.36	0.50	2.07	0.56	2.62	0.43	1.97	0.35
16	12	1–4	2.08	0.50	2.07	0.62	1.89	0.23	1.14	0.17	1.67	0.44	2.44	0.57	1.88	0.27
17	32	1–4	2.38	0.53	2.13	0.59	1.97	0.55	1.76	0.44	2.34	0.57	2.49	0.64	2.18	0.38
18	22	1–4	1.53	0.23	1.91	0.25	1.86	0.25	1.17	0.22	2.11	0.49	2.11	0.38	1.78	0.14
19	20	1–4	2.04	0.50	2.18	0.77	2.16	0.46	1.47	0.40	2.38	0.58	2.91	0.66	2.19	0.45
20	44	1–4	1.85	0.41	1.80	0.41	1.86	0.50	1.23	0.21	1.95	0.63	2.19	0.58	1.81	0.28
21	31	1–4	1.74	0.39	1.97	0.48	2.08	0.58	1.32	0.30	1.69	0.53	2.61	0.56	1.90	0.31
22	22	1–4	1.95	0.41	1.97	0.54	2.02	0.62	1.35	0.44	1.86	0.64	2.51	0.51	1.94	0.38
23	29	1–4	1.73	0.40	2.20	0.50	1.98	0.49	1.25	0.27	1.29	0.47	2.54	0.58	1.83	0.30
24	20	1–4	1.75	0.46	2.47	0.50	2.07	0.28	1.43	0.39	1.95	0.39	2.82	0.37	2.08	0.24
Total	634	1–4	1.91	0.49	2.23	0.64	2.05	0.49	1.45	0.45	1.97	0.70	2.66	0.59	2.05	0.39
			F(23,610) = 9.29***		F(23,610) = 10.11***		F(23,610) = 4.93***		F(23,610) = 10.10***		F(23,610) = 11.52***		F(23,610) = 7.56***		F(23,610) = 14.31***	

^*p < 0.05; **p < 0.01; ***p < 0.001. CI: Clinical Integration, PI: Professional Integration, OI: Organizational Integration, FI: Functional Integration, SI: System Integration, NI: Normative Integration, TI: Total Integration.

**Figure 2:**
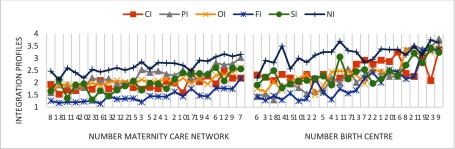
Integration profiles of Maternity Care Networks and birth centres (sorted by total integration score).* *CI: Clinical Integration, PI: Professional Integration, OI: Organizational Integration, FI: Functional Integration, SI: System Integration, NI: Normative Integration.

Between-subgroup post-hoc comparisons showed statistically significant differences Maternity Care Networks for all items, dimensions and the total integration scores. The highest scores and the lowest scores differ more than one point on the professional, functional, system and normative dimensions of integration (on a scale of one to four). The largest differences are on the professional dimension (1.44). For all Maternity Care Networks the mean scores on normative integration are the highest and on functional integration the lowest of all dimensions. For most of the networks (63%) professional integration is second highest. (see Figure 2).

The advisors working with organizations familiar with the Maternity Care Networks nominated Maternity Care Network 7 as the most integrated network and Maternity Care Networks 5 and 8 were described as the least integrated. Their evaluation is in line with our analysis: Maternity Care Network 7 had the highest mean total integration score and Maternity Care Network 8 the lowest. The score of Maternity Care Network 5 was only slightly higher. During the information meetings, participants usually recognised their own results. If not, the networks discussed their results during the meetings. It turned out that while some community midwives are involved in one Maternity Care Network others participate in more than one because they are practicing in a region with more than one hospital and the Maternity Care Networks are formed around hospitals. Community midwives who are actively participating in a particular network are usually more familiar with the organization of that network than community midwives who are more distant from its daily practise.

The reliability of the total MCN-IQ showed a Cronbach’s alpha of 0.82, showing good internal consistency (see Table 4). Within each dimension, Cronbach’s alpha ranged from 0.14–0.66, suggesting there is low internal consistency between items within the dimensions. Furthermore, we observed that in some Maternity Care Networks the range of mean scores on the integration dimensions varied more than two points, caused mostly by a difference in answers given by community and clinical midwives. The differences on the mean scores of all dimensions between primary and secondary care professionals were relatively small.

**Table 4:** Mean, SD, Range and Cronbach’s α for each dimension of integration for MCN-IQ and DBC-IQ.

Dimension	MCN-IQ (n = 634)					DBC-IQ (n = 56)				

	Number of items	Mean	SD	Range	Cronbach’s α	Number of items	Mean	SD	Range	Cronbach’s α

**Clinical integration**	4	1.91	0.49	2.50	0.44	4	2,59	0,47	1,75	0.53
**Professional integration**	4	2.23	0.64	3.00	0.55	4	2,82	0,77	3,00	0.53
**Organizational integration**	3	2.05	0.49	2.50	0.36	4	2,68	0,70	2,75	0.63
**Functional integration**	3	1.45	0.45	2.33	0.40	4	2,31	0,76	2,75	0.62
**System integration**	2	1.97	0.70	3.00	0.14	4	2,54	0,47	2,00	0.28
**Normative integration**	4	2.66	0.59	2.75	0.66	4	3,41	0,48	1,75	0.62
**Total**					0.82					0.86

### Dutch Birth Centre Study: DBC-IQ

The average item missing rate of the DBC-IQ was 5% (66 of 1326 items). Maximum item missing rates per dimension ranged from 5.2 to 13.8%. The highest missing rates were on the functional and system dimensions (see Table 2).

Table 5 reports the mean scores for each of the dimensions of integration and the total integration scores.

**Table 5:** Integration characteristics of birth centres.

Dutch Birth Centre Study																
			CI		PI		OI		FI		SI		NI		TI	
			
BC	N	Range	M	SD	M	SD	M	SD	M	SD	M	SD	M	SD	M	SD

1	2	1–4	2.83	0.24	3.38	0.18	2.88	0.18	2.50	0.35	2.70	0.14	4.00	0.00	3.05	0.07
2	1	1–4	2.25		2.00		1.50		3.33		2.33		3.50		2.49	
3	4	1–4	2.19	0.38	2.06	0.72	2.00	0.41	1.56	0.38	2.65	0.47	3.31	0.77	2.30	0.45
4	2	1–4	2.13	0.18	3.13	0.53	3.13	0.18	1.63	0.18	2.40	0.28	4.00	0.00	2.73	0.16
5	3	1–4	2.67	0.29	2.33	0.95	2.50	1.09	2.17	0.63	2.73	0.46	3.75	0.43	2.69	0.62
6	1	1–4	2.75		2.75		2.50		2.00		2.60		3.75		2.73	
7	3	1–4	2.83	0.29	2.36	0.13	1.92	0.38	2.11	0.54	2.47	0.31	2.92	0.38	2.43	0.09
8	3	1–4	2.50	0.25	3.42	0.14	3.42	0.29	2.56	0.51	2.62	0.20	3.25	0.50	2.96	0.21
9	3	1–4	3.17	0.58	3.58	0.72	3.50	0.66	3.25	0.43	3.07	0.58	3.50	0.43	3.34	0.57
10	3	1–4	2.08	0.58	2.58	0.29	2.58	0.38	1.50	0.66	2.33	0.12	3.25	0.66	2.39	0.34
11	2	1–4	2.13	0.18	2.50	1.06	2.71	1.47	2.13	0.53	2.70	0.99	3.88	0.18	2.67	0.68
12	3	1–4	2.42	0.29	2.08	0.14	2.92	0.63	1.42	0.52	2.18	0.55	3.17	0.29	2.36	0.30
13	2	1–4	2.75	0.00	3.00	0.94	3.13	0.18	2.38	0.53	2.71	0.06	3.50	0.35	2.91	0.34
14	2	1–4	2.50	0.35	2.50	0.71	1.88	0.18	1.88	0.88	1.98	0.32	4.00	0.00	2.45	0.18
15	2	1–4	2.13	0.18	2.88	0.88	2.63	0.88	1.88	1.24	2.10	0.71	2.88	0.53	2.41	0.74
16	2	1–4	3.00	0.00	3.25	0.71	2.63	0.88	3.13	0.53	2.35	0.21	3.46	0.29	2.97	0.44
17	2	1–4	2.38	0.88	3.13	0.53	2.63	0.53	1.88	0.18	2.30	0.14	3.38	0.18	2.61	0.06
18	2	1–4	2.25	0.35	1.54	0.29	2.00	0.35	2.38	0.88	2.88	0.18	3.13	0.53	2.36	0.10
19	5	1–4	3.10	0.14	3.75	0.31	3.25	0.56	3.35	0.29	2.61	0.56	3.25	0.40	3.22	0.19
20	3	1–4	2.97	0.46	2.50	0.66	2.33	0.29	2.56	0.10	2.34	0.59	3.44	0.27	2.69	0.31
21	1	1–4	2.00		3.00		2.75		2.50		3.00		3.50		2.79	
22	3	1–4	2.75	0.50	2.83	0.88	2.58	0.52	2.08	0.88	2.13	0.31	2.92	0.63	2.55	0.57
23	2	1–4	2.75	0.35	3.75	0.00	3.38	0.18	3.13	0.18	3.40	0.28	3.75	0.00	3.36	0.07
Total	56	1–4	2.59	0.47	2.82	0.77	2.68	0.70	2.31	0.76	2.54	0.47	3.41	0.48	2.73	0.45
			F(22,35) = 1.80		F(22,35) = 2.13*		F(22,35) = 2,08*		F(22,35) = 5.58***		F(22,35) = 1.38		F(222,35) = 1.21		F(22,35) = 1.81	

^*p < 0.05; **p < 0.01; ***p < 0.001. CI: Clinical Integration, PI: Professional Integration, OI: Organizational Integration, FI: Functional Integration, SI: System Integration, NI: Normative Integration, TI: Total Integration.

Post-hoc comparisons identified statistically significant differences between birth centres for the professional, organizational and functional dimensions of integration and on 50% of the items. The highest scores and the lowest scores of birth centres differed by two or more points on the professional and organizational dimensions of integration and by more than one point on the other dimensions (on a scale from one to four). The largest differences between these birth centres were on the professional dimension (2.21). For 82% of the centres, the mean scores on the normative dimension were the highest of all dimensions. The functional dimension had the lowest scores for 48% of centres, and system integration had the lowest scores for 26% of the centres (see Figure 2).

The reliability of the total DBC-IQ showed a Cronbach’s alpha of 0.86, showing good internal consistency (see Table 4). Within the dimensions, Cronbach’s alpha ranged from 0.28–0.63, suggesting there is low internal consistency between items within one dimension. The lowest alpha was on the system dimension, the highest on the organizational dimension. In two birth centres (8%), the range of mean scores between respondents was larger than two points on one dimension of integration. For eight other birth centres (35%), we found the range of mean scores to be between one and two points.

## Discussion

This study examined the feasibility, discriminative validity, and reliability of the Maternity Care Network and Dutch Birth Centre Integration Questionnaires. We have shown that both questionnaires are feasible for the evaluation of integration in Maternity Care Networks or birth centres. The questionnaires show acceptable average missing rates according to the literature [30,31]. These rates are higher for the MCN-IQ than for the DBC-IQ, just like the mean percentage of maximum missing items. For both questionnaires, highest missing rates were assessed at the functional dimension, which may have been caused by the diversity of the respondents. In the Dutch Birth Centre Study, only respondents who were very familiar with the birth centre were invited to participate. In contrast, in the Maternity Care Network Study, all participants who were in some way connected to the Maternity Care Network were invited. Even professionals hardly involved and, therefore, unfamiliar with the organization of the network filled in the questionnaire. This also could explain the relatively high percentage of respondents with more than 30% missing answers. For future use of the questionnaire, we recommend that only respondents who are at least moderately familiar with the organization of birth care in the region are invited to complete the questionnaire.

Both questionnaires are able to discriminate between Maternity Care Networks and birth centres based on the level of integration. We observed statistically significant differences between Maternity Care Networks on all items and dimensions of integration and the total integration score. Between birth centres, we only observed statistically significant differences on the professional, organizational and functional dimensions of integration. The distinctive integration profiles of Maternity Care Networks and birth centres as presented showed similar patterns with highest scores on normative integration followed by professional and organizational integration, and lowest scores on clinical and functional integration (see Figure 2). This pattern is particularly noticeable in the Maternity Care Network profiles, but also recognizable in the birth centres. These findings are consistent with theories about the development of collaborative groups. Integration is to a large extent based on professional behaviour and attitude. Informal coordination mechanisms based on culture, shared values and vision are essential primary conditions towards integration on a professional and organizational level [10,33]. Normative integration has to be implemented first before realizing better integration at the professional and organizational levels. Patient-centred care (clinical integration) is a key concept of integrated care but it demands a change in focus in organizations that are traditionally more physician-centred [2].

We did observe differences between the MCN-IQ and DBC-IQ in distinctiveness. This may be caused by the dissimilarity in the number of respondents that completed in the questionnaire, because significance depends on the size of the differences and the sample size. We also noticed a dissimilarity in the differences between the highest and lowest scoring Maternity Care Networks and birth centres on the dimensions of integration. In the Maternity Care Network study, these differences were smaller than in the Dutch Birth Centre Study. A possible explanation for this dissimilarity is selection bias in the Maternity Care Network Study. We included a self-selected group of Maternity Care Networks, namely those who were already interested in the issue and requested meetings to learn more about integrated birth care. It is possible that more integrated Maternity Care Networks were less interested in such information meetings, because they already had their own information about integrated birth care. Maternity Care Networks that were less integrated were probably also not interested in these meetings, perhaps because they did not see the added value of such meetings. It is conceivable that networks that participated in this study were all, more or less, at the same stage of their integration process, which could explain the small differences. This possibility of our having included only a select group of Maternity Care Networks in the study is in contrast to the Dutch Birth Centre Study, where all birth centres participated.

We determined that for both questionnaires the internal consistency was good. This indicates that the items and dimensions as a whole are coherent; they all contribute to the same overall concept of integration. However, we observed a low internal consistency between items within each dimension for both questionnaires, especially for the MCN-IQ, indicating that items within each dimension are not, or are only weakly, correlated with each other. In this questionnaire, only the normative dimension showed a reasonable internal consistency. These findings indicate that there is no psychometric consistency within the items of one dimension. It confirms the basic principle of the development of the taxonomy. In our view this finding confirms the underlying key feature of the six dimensions of integration – a range of partly unrelated determinants within one dimension, all contributing to that dimension. The separate dimensions may be regarded as clinimetric scales, often used to describe the clinical condition of a patient (for example the Apgar Score, consisting of predictors of a neonatal condition that are uncorrelated with one another [32]. Further research is necessary to investigate whether our findings can be confirmed in other settings (both in the Netherlands and in other countries).

We observed that in some Maternity Care Networks the mean scores on the integration dimensions varied between respondents within one network. The explanation for this variation may be that community midwives are the largest number of professionals within the Maternity Care Networks, making differences within one group more plausible. However, it is also conceivable that community midwives are involved in the Maternity Care Networks in varying degrees, depending on their local situation. Despite the wide range in answers, we found that the differences on the mean scores of all dimensions between primary and secondary care professionals in the Maternity Care Networks were relatively small. This indicates that the range in answers is rather similar in those groups of professionals. In the Dutch Birth Centre Study, respondents not only were smaller in number, they were also from similar disciplines (community midwives, managers of birth centres and maternity care assistants).

### Limitations

By interpreting the results, limitations of the study should be considered. First, the psychometric properties of the questionnaires are examined only in the Netherlands. Because of the specific key features of the Dutch maternity care system (for example independent practicing community midwives and community midwives as gatekeepers to secondary obstetric care), it is yet unclear whether the questionnaires can be used in other maternity care systems. We assume that the questionnaires can be used in other countries with a different maternity care system. Using and testing the questionnaires in other countries could contribute to a higher external validity. Also, the number of respondents that filled in the questionnaires in the Dutch Birth Centre Study is too small to perform a valuable validation. Using and testing the DBC-IQ in more birth centres also in other countries, will improve its validity. Another limitation relates to the respondents who filled in the questionnaires. In our study, only data from a health care provider and manager perspective are collected. Reflections from a client’s perspective are lacking. Because the multidimensional aspects of integration in which patient centeredness (clinical integration) is an important key feature, this perspective should also be included in an assessment to get a multidimensional view of integration. At last, we only tested the validity of the questionnaires for assessment of the level of integration between different sites. A next step will be to explore the relation between level of integration, outcomes of care, client experiences, and costs. Further research is necessary to test whether the questionnaires are able to assess changes in levels of integration over time.

### Implications for practice and research

This study tested a newly developed instrument to assess aspects of integration in a maternity care system and contributes to a better understanding of integrated care in these settings. Using the instruments gives us an opportunity to compare *relative* levels of integration (between different sites and from different perspectives), but we do not know if the instrument is also usable to measure the *absolute* degree of integration. Professionals could have different interpretations of integration and its levels. The complexity of integrated care makes it difficult to test this: there is no ‘golden standard’ of levels of integration. Therefore, the instrument might be useful by comparing outcomes of care, related to differences in levels of integration, in different sites, but less useful in judging the levels of integration of an individual site. In the Dutch Birth Centre Study we tried to tackle the problem of different interpretations of integration by combining the questionnaires with personal interviews, conducted by one researcher. Further research is necessary to explore whether this solution solves this problem.

Although evidence is available on the effectiveness of integrated care in chronic care, until now, there is no evidence for this assumption in birth care, even while current government policy in the Netherlands is based on it. For example, beginning in 2017, the payment system for maternity care will allow the bundling of payments for both primary and secondary birth care providers, a change that will require more integration between both echelons. Using the questionnaires might be a valuable contribution to examine the assumption that integrated birth care improves quality of care by combining integration profiles and perinatal outcomes, client experiences and costs. When using them in further research, these data could be used to explore the effectiveness of integrated birth care. Also these data can be used to explore whether the integration questionnaires are able to predict effectiveness of a birth care setting.

In our view, the instrument can also be used to support health care professionals, managers, policymakers and health insurance companies involved in the organization of integrated birth care, allowing them to better understand its concepts, which might, in turn, help the political debate. However, based on present studies, we find the instrument unsuitable as management tool for, for example, health insurance companies. Further research is necessary to explore this application.

## Conclusion

The MCN-IQ and DBC-IQ are feasible and can discriminate between Maternity Care Networks and birth centres with different integration profiles in the Netherlands. The questionnaires offer an opportunity to better understand integrated care as an approach to the delivery of health services in different models of integrated birth care. Further research is necessary to explore whether the instruments can be applied in other countries and whether they can be used to assess changes in levels of integration over time, to measure absolute levels of integration and to predict outcome of a birth care setting. The development of the questionnaires is one more step in building knowledge of the complexity of integrated care.
